# Intraclonal Enrichment of IL-23 Receptor Complex Expression in the Proliferative Fraction of Chronic Lymphocytic Leukemia

**DOI:** 10.3390/ijms27031202

**Published:** 2026-01-25

**Authors:** Martina Cardillo, Fabiana Ferrero, Nadia Bertola, Ennio Nano, Rosanna Massara, Maria Cristina Capra, Daniele Reverberi, Monica Colombo, Vanessa Cossu, Fabio Ghiotto, Adalberto Ibatici, Emanuele Angelucci, Antonino Neri, Massimo Gentile, Fortunato Morabito, Andrea Nicola Mazzarello, Manlio Ferrarini, Franco Fais, Giovanna Cutrona

**Affiliations:** 1 Department of Experimental Medicine, University of Genoa, 16132 Genoa, Italy; mcardillo1@northwell.edu (M.C.); fabiana.ferrero@edu.unige.it (F.F.); vanessa.cossu@edu.unige.it (V.C.); fabio.ghiotto@unige.it (F.G.); andreanicola.mazzarello@edu.unige.it (A.N.M.); 2 Molecular Pathology Unit, IRCCS Azienda Ospedaliera Metroplitana, 16132 Genoa, Italy; nadia.bertola@hsanmartino.it (N.B.); ennio.nano@hsanmartino.it (E.N.); rosanna.massara@hsanmartino.it (R.M.); mariacristina.capra@hsanmartino.it (M.C.C.); daniele.reverberi@hsanmartino.it (D.R.); monica.colombo@hsanmartino.it (M.C.); giovanna.cutrona@hsanmartino.it (G.C.); 3 Hematology and Cellular Therapy Unit, IRCCS Azienda Ospedaliera Metroplitana, 16132 Genova, Italy; adalberto.ibatici@hsanmartino.it (A.I.); emanuele.angelucci@hsanmartino.it (E.A.); 4 Scientific Directorate, Azienda USL-IRCCS di Reggio Emilia, 42122 Reggio Emilia, Italy; antonino.neri@ausl.re.it; 5 Hematology Unit, Department of Onco-Hematology, AO of Cosenza, 87100 Cosenza, Italy; m.gentile@aocs.it; 6 Department of Pharmacy, Health and Nutritional Science, University of Calabria, 87036 Rende, Italy; 7 Gruppo Amici Dell’Ematologia Foundation-GrADE, 42100 Reggio Emilia, Italy; f.morabito53@gmail.com

**Keywords:** chronic lymphocytic leukemia, IL-12 receptor complex, IL-23 receptor complex, TLR-9, antigen-independent stimulation

## Abstract

Chronic lymphocytic leukemia (CLL) is a dynamic malignancy in which intraclonal subfractions differ in activation history and responsiveness to microenvironmental signals. Here, we investigated the expression and inducibility of IL-12 family receptor subunits (IL-23R, IL-12Rβ1, IL-12Rβ2) and the related receptor complexes in recirculating CLL cells, with a focus on CXCR4/CD5-defined fractions: the proliferative fraction (PF; CXCR4^dim^/CD5^bright^; most recently divided, tissue-emigrated cells) and the resting fraction (RF; CXCR4^bright^/CD5^dim^; older, quiescent cells). At baseline, IL-12Rβ1 was enriched in the PF and was associated with a higher proportion of cells expressing IL-23R and IL-12R receptor complexes. Concomitantly, RT-qPCR disclosed higher IL-12Rβ1 mRNA levels. Following antigen-independent activation with CpG or CpG + IL-15, there was a marked increase in IL-23R and IL-12Rβ1 but not in IL-12Rβ2 surface expression, resulting in preferential upregulation of the IL-23R complex over the IL-12R complex. Fraction-specific analyses showed stronger induction of IL-23R and IL-23R complex expression in PF compared with RF. These findings identify an intraclonal bias toward IL-23 responsiveness in the CLL cells with a phenotype of recently divided, tissue-emigrated cells and suggest the IL-23/IL-23R axis as a potential therapeutic target.

## 1. Introduction

Chronic lymphocytic leukemia (CLL) is the most common adult leukemia in Western countries and is characterized by the clonal accumulation of mature CD5^+^ B lymphocytes in the peripheral blood, bone marrow, and secondary lymphoid organs [1]. Early studies emphasized that CLL pathogenesis is strongly shaped by chronic antigenic stimulation and by B cell receptor (BCR) biology, which plays a central role in determining leukemic cell survival and clonal evolution [2,3]. Subsequent work further redefined CLL as a dynamic disease in which measurable leukemic cell turnover and continuous trafficking between lymphoid tissues and peripheral blood, rather than passive accumulation alone, contribute to disease progression [4]. Although many patients experience an indolent clinical course and may not require therapy for years, a significant subset exhibits aggressive disease requiring early treatment. This marked clinical heterogeneity reflects both intrinsic features of leukemic clones and extrinsic signals provided by the tumor microenvironment, which is pivotal for sustaining survival, activation, and proliferation of CLL cells [5,6,7].

Beyond interpatient variability, CLL displays marked intraclonal heterogeneity. The leukemic population comprises subfractions with distinct proliferative histories and biological properties [8]. A well-established framework to dissect this complexity relies on the reciprocal surface expression of CD5 and the chemokine receptor CXCR4, which identifies CLL subpopulations differing by time since last division. CXCR4^dim^/CD5^bright^ cells represent the most recently divided, tissue-emigrated cells (proliferative fraction, PF), whereas CXCR4^bright^/CD5^dim^ cells correspond to older, quiescent cells (resting fraction, RF). Intermediate fractions reflect transitional stages in this trafficking cycle [9]. These subpopulations differ not only phenotypically but also functionally, displaying distinct transcriptional programs, telomere length, activation markers, and sensitivity to microenvironment-derived signals. Importantly, the CXCR4^dim^/CD5^bright^ fraction is enriched for cells recently stimulated in proliferation centers within lymph nodes, suggesting heightened susceptibility to additional activating signals upon re-entry into supportive niches.

Among microenvironmental mediators influencing CLL biology, cytokines of the interleukin-12 (IL-12) family play key roles in immune regulation and inflammation [10]. IL-12 signals through a heterodimeric receptor composed of the IL-12Rβ1 and IL-12Rβ2 subunits, whereas IL-23 signals via a receptor consisting of IL-12Rβ1 paired with IL-23R [11,12]. Functionally, IL-12 typically promotes Th1 differentiation and cytotoxic immune responses [13], whereas IL-23 supports Th17 polarization, bridges innate and adaptive immunity, and sustains chronic inflammatory responses [14].

In normal B cell physiology, expression of the IL-23 receptor complex has been detected in early B lymphocytes, germinal center B cells, and plasma cells but is usually expressed at low or undetectable levels in mature circulating B cells [15]. In malignant contexts, IL-23R/IL-23 biology has been investigated in childhood B-acute lymphoblastic leukemia, B cell lymphomas, and in plasma-cell neoplasia [16,17,18].

In CLL, our group previously showed that circulating leukemic cells frequently exhibit an incomplete IL-23 receptor phenotype, characterized by IL-23R expression in the absence of IL-12Rβ1. CD40/CD40L interactions provided by activated T cells can induce expression of the complete IL-23 receptor complex, enabling responsiveness to IL-23 and supporting autocrine/paracrine signaling loops [19]. Importantly, these events occur independently of BCR engagement, underscoring the relevance of non-antigen-driven microenvironmental signals in shaping cytokine responsiveness. In contrast, IL-12 has been associated with growth-inhibitory and tumor-suppressive effects. Genetic IL-12Rβ2 deficiency predisposes murine models to autoimmunity and spontaneous lymphoid malignancies, highlighting a protective role for IL-12 signaling in lymphoid homeostasis [20]. Consistently, reduced IL-12Rβ2 expression has been reported in several hematologic malignancies, suggesting selective pressure against intact IL-12 signaling during tumor evolution [16,17,18].

Given the limited understanding of how IL-12 family receptors are distributed within circulating CLL clones across distinct trafficking/proliferation states, this study aimed to (i) define, in ex vivo recirculating CLL cells, the expression of IL-23R, IL-12Rβ1, and IL-12Rβ2 across the main CXCR4/CD5-defined fractions, with particular focus on the resting fraction (RF) and proliferative fraction (PF); (ii) quantify fraction-specific co-expression patterns consistent with assembly of the IL-23 receptor complex (IL-23R + IL-12Rβ1) versus the IL-12 receptor complex (IL-12Rβ1 + IL-12Rβ2); and (iii) determine whether, and to what extent, these receptor subunits/complexes are inducible by antigen-independent activation, using TLR9 stimulation (CpG) with or without IL-15, both in bulk CLL cells and in a fraction-resolved manner, to test for an intraclonal bias toward the IL-23/IL-23R axis relative to IL-12/IL-12R.

We therefore examined IL-12 family receptor subunit expression and co-expression in recirculating CLL cells ex vivo and after antigen-independent stimulation.

## 2. Results

**Figure 1:**
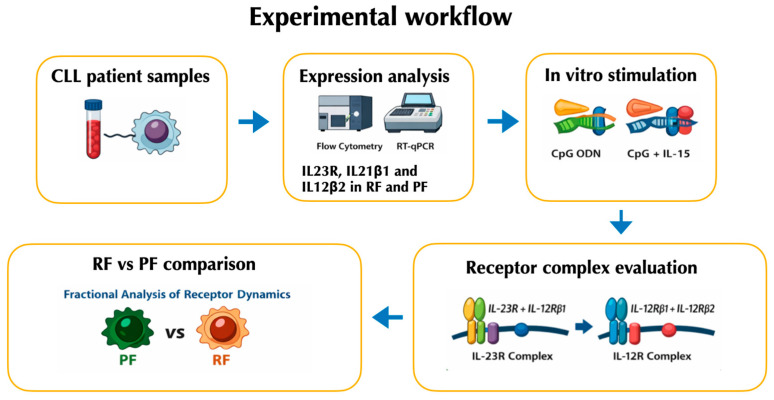
Schematic overview of the experimental workflow described in the Results Section.

The workflow of the results has been summarized in Figure 1.

### 2.1. The Proliferative Fraction Shows Higher Expression of IL-23R and IL-12R Receptor Complexes in Recirculating CLL Cells

CLL samples were retrieved from a frozen cell bank and were obtained from patients followed at our institutions. All patients were treatment-naïve at the time of sampling. Details are reported in Materials and Methods and in Table S1.

**Figure 2:**
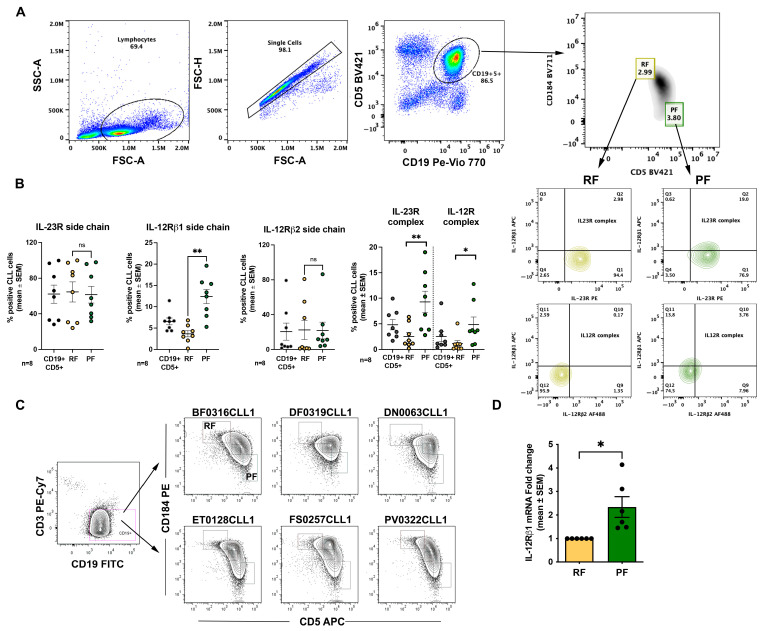
IL-23R and IL-12R expression in ex vivo CLL cells assessed by flow cytometry. FSC is proportional to cell size and SSC reflects intracellular complexity/granularity (each dot represents one cell). Axes in marker plots show fluorescence intensity for the indicated markers. Percentages within gates indicate the fraction of cells relative to the parent population. Lymphocytes were identified by FSC/SSC properties, followed by doublet exclusion using pulse height vs. width plots. Within the single-cell gate, CD19^+^/CD5^+^ CLL cells were selected. This population was further sub-divided into RF and PF based on the differential expression of CD184 and CD5. (**A**) Gating strategy used to identify lymphocytes, single cells, CD19^+^ CLL cells, and to discriminate proliferative fraction (PF) and resting fraction (RF); (**B**) surface expression of IL-23R, IL-12Rβ1, and IL-12Rβ2 receptor subunits in ex vivo CLL cells, shown for CD19^+^ PF and RF populations; (**C**) representative flow cytometry plots showing sorting of PF and RF populations from six CLL samples according to the gates indicated; (**D**) IL-12Rβ1 mRNA expression in sorted RF and PF cells, measured by RT-qPCR. Data are shown as mean ± SEM. Statistical significance of the difference is evaluated using the two-sided Wilcoxon signed-rank test. * *p* < 0.05; ** *p* < 0.01, ns: not significant. FSC: forward scatter; SSC: side scatter; PE Phycoerythrin; PE-Cy7: Phycoerythrin–Cyanine 7; APC: Allophycocyanin; AF 488: Alexa Fluor 488; PE VIO 770: near-infrared fluorochrome emitting in the ~770–780 nm range.

We first analyzed expression of the IL-23R and IL-12R receptor complexes across phenotypically distinct CLL fractions defined according to Calissano et al. [9] (see also Introduction). In our flow cytometry analyses, data are presented as the percentage of positive cells; however, in this experimental setting this readout also provides an indirect proxy for changes in mean fluorescence intensity, because shifts in signal intensity translate into changes in the fraction of events exceeding the positivity threshold. Thus, receptor complexes were quantified as double-positive populations for the relevant subunits (IL-23R^+^IL-12Rβ1^+^ for the IL-23R complex and IL-12Rβ1^+^IL-12Rβ2^+^ for the IL-12R complex). The gating strategy used to identify these fractions by flow cytometry is shown in Figure 2A. For analysis of IL-23R and IL-12R expression, we focused on RF and PF.

When analyzing individual receptor subunits, we observed a significant difference between RF (3.7 ± 0.7% mean and SEM) and PF (12.4 ± 1.7% mean and SEM) only for IL-12Rβ1 (Figure 2B). However, when receptor complexes were considered, PF displayed a higher percentage of cells co-expressing the IL-23R complex (2.5 ± 0.8% vs. 9.3 ± 2% mean and SEM) and the IL-12R complex (1.1 ± 0.6% vs. 4.8± 1.5%). Given the higher representation of IL-12Rβ1 at the cell surface, we evaluated IL-12Rβ1 mRNA levels by RT-qPCR in sorted RF and PF populations. Consistent with the flow cytometry data, this analysis confirmed higher IL-12Rβ1 expression in PF (Figure 2C,D).

### 2.2. CpG + IL-15 Stimulation Induces IL-23R and IL-12R Receptor Complexes in CLL Cells

We next investigated whether expression of the IL-23R complex and the IL-12R complex could be induced in CLL cells following TLR9 activation. Figure 2B shows that, in line with previous observations [19], the IL-23R complex was detectable only in a low percentage of ex vivo CLL cells. At the whole-clone level, the IL-12R complex was generally expressed in a relatively low percentage of cells, with few exceptions, primarily due to limited expression of the IL-12Rβ2 subunit (Figure 2B).

**Figure 3:**
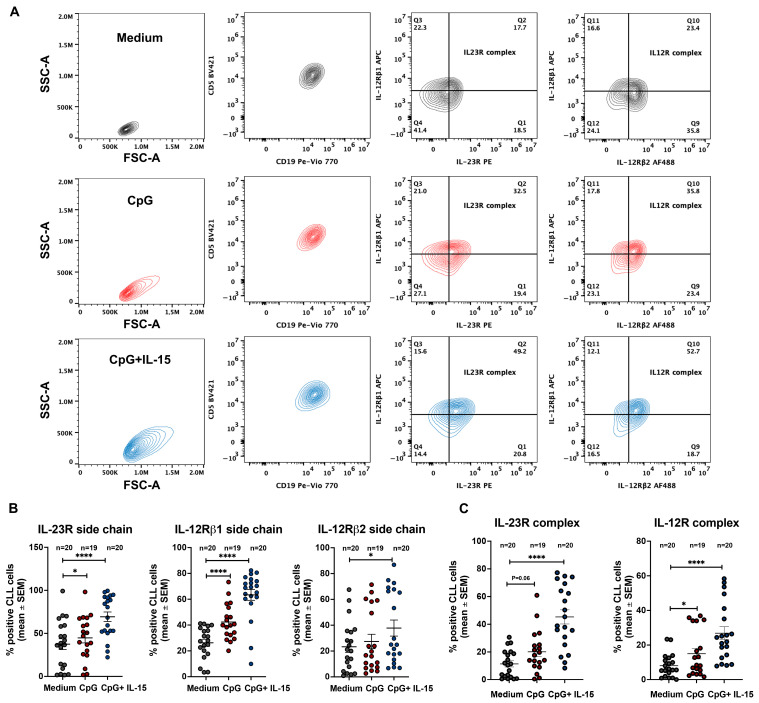
IL-23R and IL-12R expression in CLL cells after incubation with CpG and CpG + IL-15 for 72 h assessed by flow cytometry analyses. (**A**) Representative flow cytometry gating strategy used to identify CLL cells and to assess IL-23R and IL-12R complex expression under the indicated culture conditions (medium alone, CpG, or CpG + IL-15) after 72 h; (**B**) percentage of CLL cells expressing the IL-23R receptor subunit, IL-12Rβ1 receptor subunit, and IL-12Rβ2 receptor subunit following 72 h incubation under the indicated conditions; (**C**) percentage of CLL cells expressing the IL-23R complex and the IL-12R complex following 72 h incubation under the indicated conditions. Statistical significance of the difference is evaluated using the two-sided Wilcoxon signed-rank test. * *p* < 0.05; **** *p* < 0.0001. FSC: forward scatter; SSC: side scatter; PE Phycoerythrin; PE-Cy7: Phycoerythrin–Cyanine 7; APC: Allophycocyanin; AF 488: Alexa Fluor 488.

To activate leukemic B cells, purified CLL cells from 20 samples were cultured for 72 h with CpG alone or in combination with IL-15, a cytokine known to synergize with CpG in promoting CLL cell survival and proliferation [21]. CpG + IL-15 was generally more efficient in inducing a significant upregulation of IL-23R (CpG + IL-15 69.4 ± 5.5%vs Medium 37.5 ± 6% mean and SEM, *p* < 0.0001) and IL-12Rβ1(CpG + IL-15 63.4 ± 4% vs. Medium 26.3 ± 2.7% mean and SEM, *p* < 0.0001) surface expression. In contrast, IL-12Rβ2 (CpG + IL-15 37.8 ± 6.2 vs. medium 23.3 ± 4.3% mean and SEM, *p* = 0.04) upregulation was detected only in a minority of samples (Figure 3B) and did not yield a significant overall increase.

After 72 h of stimulation, both receptor complexes were detectable; however, IL-23R complex expression was consistently detected in a higher percentage of CLL cells (CPG + IL-15 45.3 ± 5% vs. medium 11.4 ± 2.2% mean and SEM, *p* < 0.0001) than the IL-12R complex (CPG + IL-15 26.8 ± 3.7% vs. medium 8 ± 1.5% mean and SEM, *p* < 0.0001, Figure 3C). This preferential induction of the IL-23R complex largely reflected the limited induction of the IL-12Rβ2 subunit. Nevertheless, IL-12R complex expression was higher in CpG + IL-15–stimulated CLL cells than in unstimulated cells.

### 2.3. CpG + IL-15 Stimulation Differentially Upregulates IL-23R and IL-12R Receptor Subunits in Resting and Proliferative Fractions

**Figure 4:**
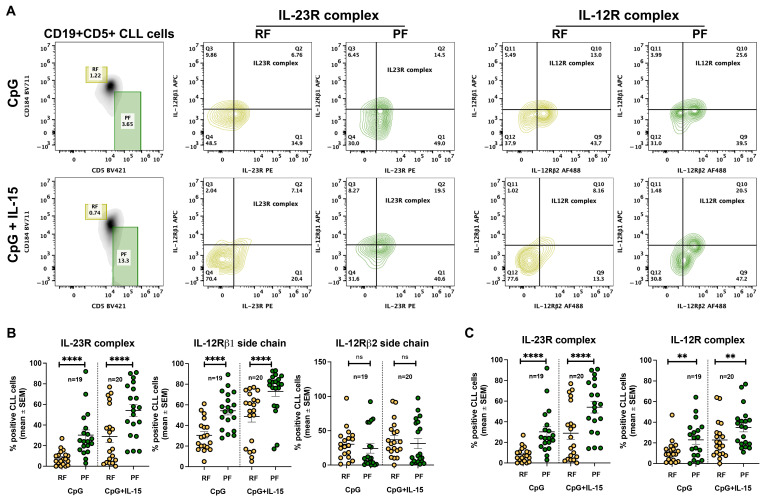
Differential expression of IL-23R and IL-12R in CLL RF and PF subsets after stimulation with CpG and CpG + IL-15 assessed by flow cytometry analyses. (**A**) Representative flow cytometry gating strategy used to identify CLL cells and to distinguish RF and PF subsets, followed by assessment of IL-23R and IL-12R complex expression after 72 h incubation with CpG or CpG + IL-15; (**B**) percentage of RF and PF CLL cells expressing the IL-23R receptor subunit, IL-12Rβ1 receptor subunit, and IL-12Rβ2 receptor subunit after 72 h incubation under the indicated conditions; (**C**) percentage of RF and PF CLL cells expressing the IL-23R complex and the IL-12R complex after 72 h incubation under the indicated conditions. Each symbol represents an individual CLL sample; horizontal bars indicate mean ± SEM. Statistical significance of the difference is evaluated using the two-sided Wilcoxon signed-rank test. ** *p* < 0.01; **** *p* < 0.0001, ns: not significant. FSC: forward scatter; SSC: side scatter; PE Phycoerythrin; PE-Cy7: Phycoerythrin–Cyanine 7; APC: Allophycocyanin; AF 488: Alexa Fluor 488; BV421 = Brilliant Violet 421.

We then assessed whether cells expressing IL-23R and IL-12R complexes differed between RF and PF following CpG + IL-15 stimulation. The gating strategy used to identify these fractions is shown in Figure 4A and overlaps with that applied to ex vivo CLL cells. Using this approach, IL-23R and IL-12R receptor subunit expression was analyzed in RF and PF after 72 h of stimulation.

The percentage of IL-12Rβ1-positive cells increased in both fractions but remained consistently higher in PF. IL-12Rβ2 expression remained largely unchanged across fractions, showing only a modest increase in RF. In contrast, IL-23R expression was markedly induced by stimulation in both RF and PF, with a significantly higher percentage of IL-23R^+^ cells detected in PF compared with RF (Figure 4B).

Analysis of the complete receptor complexes further supported these findings. IL-12R complex expression did not differ significantly between RF and PF under control, CpG, or CpG + IL-15 conditions. By contrast, IL-23R complex expression was significantly higher in PF compared with RF following CpG + IL-15 stimulation (Figure 4C).

## 3. Discussion

This study investigated the expression of IL-23R, IL-12Rβ1, and IL-12Rβ2 in recirculating CLL cells and in intraclonal subfractions defined by CD5 and CXCR4 co-expression (Figure 2A). We also assessed whether expression of these receptors could be modulated by antigen-independent stimulation (CpG alone or CpG + IL-15). Analyses were performed on whole CLL cell populations and within CD5/CXCR4-defined subfractions. We focused primarily on RF and PF, as these fractions are best characterized and were the most informative in our dataset. Thus, the intermediate fraction was not included in representative figures. 

IL-12 family receptor expression was heterogeneous across CD5/CXCR4-defined intraclonal subfractions. Notably, IL-12Rβ1 was detected in a significantly higher percentage of PF cells (Figure 2B), and RT-qPCR analysis in sorted RF and PF confirmed higher IL-12Rβ1 mRNA levels in PF (Figure 2C,D). Consistent with these findings, PF also displayed a higher proportion of cells co-expressing the IL-23R and IL-12R receptor complexes compared with RF (Figure 2B). Overall, these data indicate that IL-12Rβ1 availability is a key determinant of IL-23R and IL-12R complex assembly within recirculating CLL cells, and that its enrichment in PF may contribute to a phenotype more permissive to cytokine-driven clonal expansion.

We further show that stimulation with CpG + IL-15 induces IL-12 family receptor components (Figure 3B). After 72 h of in vitro exposure, surface IL-23R and IL-12Rβ1 increased markedly, whereas IL-12Rβ2 remained largely unchanged (Figure 3B). This pattern resulted in a robust increase in the IL-23R receptor complex and a more limited increase in the IL-12R receptor complex (Figure 3C), consistent with a cytokine-responsiveness profile skewed toward IL-23.

When receptor expression was evaluated in the context of CD5/CXCR4-defined intraclonal heterogeneity, both RF (CXCR4^bright^/CD5^dim^) and PF (CXCR4^dim^/CD5^bright^) displayed increased IL-23R and IL-12Rβ1 surface expression following stimulation (Figure 4B). However, the proportion of IL-23R complex-positive cells was higher in PF than in RF (Figure 4C). These findings suggest that the potential for IL-23 responsiveness is higher in PF-phenotype CLL cells (most recently divided, tissue-emigrated cells), as reflected by their higher proportion of IL-23R-expressing cells, whereas RF cells (older, quiescent cells) displayed a significantly lower proportion of IL-23R-expressing cells.

It is noteworthy that the most consistent induction of IL-23R and IL-12Rβ1 occurred in the presence of IL-15. In our setting, CpG alone was generally less effective at inducing significant changes in IL-23R/IL-12Rβ1 expression. This is in line with the concept that IL-15 can prevent CpG/TLR9-associated apoptosis and allow significant CLL clonal expansion [21], consistent with PF representing a fraction with higher proliferative competence. Beyond its known anti-apoptotic effects, IL-15 may also contribute to remodeling the cytokine receptor landscape of CLL cells, potentially facilitating preferential assembly of the IL-23 receptor complex in proliferative fraction cells.

The asymmetric distribution of IL-23R across intraclonal subfractions has several biological implications. First, it supports the concept that PF cells, enriched in most recently divided, tissue-emigrated cells, are particularly receptive to microenvironment-derived cytokine cues and may exploit IL-23 signaling to support expansion and survival within proliferation centers. Second, CpG/IL-15 stimulation effectively induced IL-23R complex expression (IL-23R and IL-12Rβ1) in CLL clones, and this induction was more pronounced in PF-phenotype cells.

A further relevant observation is the persistently limited induction of IL-12Rβ2 in response to CpG/IL-15 stimulation. Although recirculating CLL clones showed variable baseline IL-12Rβ2 expression, PF generally displayed a lower proportion of IL-12R complex-positive cells than IL-23R complex-positive cells, particularly after CpG/IL-15 stimulation (Figure 4C). In this setting, the limited inducibility of IL-12Rβ2 emerges as a defining constraint on IL-12 receptor complex formation, while permitting preferential engagement of IL-23-associated pathways. Of note, IL-12Rβ2 availability has been reported to influence IL-12 responsiveness in other B cell malignancies, and IL-12 family cytokine axes can be differentially configured across lymphoproliferative settings [17,18]. Beyond limiting IL-12 responsiveness, reduced IL-12Rβ2 expression has also been mechanistically linked to leukemogenesis: in murine models, genetic deficiency of IL-12Rβ2 predisposes to autoimmunity and spontaneous development of lymphoid malignancies, supporting a tumor-suppressive role for IL-12 signaling [20]. Thus, failure to adequately upregulate IL-12Rβ2 may constrain IL-12 signaling and contribute to a permissive context for leukemic persistence and progression, whereas robust induction of IL-23R supports a shift toward pro-survival, pro-inflammatory pathways. Overall, this imbalance between IL-12 and IL-23 signaling may contribute to intraclonal specialization and to the selection of subclones favoring survival and expansion.

Together, these findings support a model in which intraclonal heterogeneity in CLL is associated with a functional bias toward IL-23 responsiveness, selectively enriched in recently divided, tissue-emigrated cells with a proliferative fraction phenotype.

From a therapeutic standpoint, our findings suggest two complementary avenues. First, targeting the IL-23/IL-23R axis may disrupt pro-survival and proliferative signaling that is preferentially enriched in PF-phenotype cells. In support of this concept, we previously reported that neutralizing IL-23 can restrain disease in CLL xenograft models [19,22]. Second, approaches aimed at restoring or enhancing IL-12Rβ2 expression and signaling could re-establish tumor-suppressive IL-12 functions, counteracting the leukemogenic predisposition associated with IL-12Rβ2 deficiency. The balance between impaired IL-12Rβ2-mediated restraint and enhanced IL-23R-driven activation may therefore represent a key pathogenic mechanism in CLL biology, and future interventions could be designed to shift this equilibrium toward an anti-leukemic immune environment.

Thus, our findings are not only descriptive, but mechanistically and translationally relevant because they identify where and when IL-23 responsiveness is most likely to be functionally engaged within the CLL clone. The preferential availability/inducibility of the IL-23 receptor complex (IL-23R + IL-12Rβ1) in the proliferative fraction (PF) suggests that IL-23 signaling may be most impactful in the intraclonal compartment that is closest to proliferation centers and most exposed to microenvironmental cues, potentially sustaining survival/proliferation programs in the cells that drive clonal expansion. Conversely, the persistently limited induction of IL-12Rβ2 points to a constrained capacity to assemble the full IL-12 receptor complex, consistent with a biological skewing away from IL-12–linked restraining pathways and toward IL-23–associated programs. This imbalance supports a model in which IL-23R complex expression functions as a “permissive gate” for microenvironment-driven expansion, particularly in PF-phenotype cells.

Importantly, the IL-23 axis is already a clinically druggable pathway: monoclonal antibodies that inhibit IL-23 signaling are approved for immune-mediated inflammatory diseases (notably psoriasis/psoriatic arthritis, and for some agents inflammatory bowel disease) [23,24,25], providing extensive real-world experience on pathway inhibition and safety management. This existing therapeutic landscape strengthens the translational rationale of our results because it indicates that IL-23 pathway blockade is feasible and can be implemented with established dosing and monitoring frameworks. Moreover, target selectivity may matter in CLL: IL-23-specific p19 inhibitors (blocking IL-23 without directly blocking IL-12) could, in principle, be preferable to p40 blockade (shared IL-12/IL-23 subunit) in a context where IL-12-associated tumor-restraining effects have been proposed and where IL-12Rβ2 appears limiting.

## 4. Materials and Methods

### 4.1. CLL Cell Samples

The study was approved by the Institutional Review Boards of Northwell Health and was conducted according to the principles of the World Medical Association Declaration of Helsinki. CLL patients were diagnosed as recommended, and all subjects provided written informed consent at enrollment. In addition, part of the experiments was conducted using peripheral blood samples (n = 14) from newly diagnosed patients with Binet stage A CLL enrolled in the O-CLL1 protocol (ClinicalTrials.gov identifier NCT00917540) [26]. A total of 28 CLL patients were included in this study (see Table S1). All patients were treatment-naïve at the time of sample collection. Not all samples were used for every experimental assay due to cell number limitations; the number of patients analyzed in each experiment is indicated in the corresponding figure legends.

### 4.2. CLL Cell Isolation

CLL cells from each patient’s PB were isolated by negative selection using RosetteSep Human B Cell Enrichment Cocktail (STEMCELL Technologies, Vancouver, BC, Canada). Whole PB was incubated with the enrichment cocktail, then diluted with 2% FBS in PBS and centrifuged over RosetteSep DM-L Density Medium (STEMCELL Technologies). Purity was assessed by the Center for CLL Research. Cells were resuspended in freezing solution and cryopreserved in liquid nitrogen. Samples containing ≥95% leukemic cells were considered eligible for the study.

### 4.3. CLL Cell Culture and Stimulation

Thawed CLL cells were seeded in an enriched medium used for long-term culture of normal B cells, supplemented with insulin/transferrin/selenium (Cat. #17-838Z; Lonza, Basel, Switzerland). Notably, this medium contains 2-ME (5 × 10^−5^ M), which supports cystine-to-cysteine reduction and thereby facilitates cysteine availability for glutathione synthesis required for retained viability. Fresh medium was prepared for each experiment using stock additives. Cultures were established in 96-well round-bottom plates at 4 × 10^5^ cells per 200 µL, with duplicates for each condition. Recombinant human IL-15 (PeproTech Inc., Rocky Hill, NJ, USA) and CpG DNA TLR9 ligand (ODN-2006; InvivoGen, San Diego, CA, USA) were added to final concentrations of 15 ng/mL and 0.2 µM (1.5 µg/mL), respectively, and cultures were incubated for 72 h. Features of the CLL samples used are reported in Table S1.

### 4.4. Flow Cytometry: Cytokine Receptor Detection in Bulk CLL Cells

Live cells were identified using LIVE/DEAD Fixable Stains for flow cytometry (LIVE/DEAD™ Fixable Violet Dead Cell Stain Kit or Far-Red Dead Cell Stain Kit; Life Technologies, Carlsbad, CA, USA). For surface membrane immunofluorescence, cells (2 × 10^5^) in FACS buffer (PBS + 10% bovine serum albumin + 1% sodium azide) were incubated with primary antibodies for 20 min at 4 °C, followed by fixation with 0.1% formaldehyde in PBS. Isotype controls were processed in parallel. The following monoclonal antibodies were used: mouse anti human IL-23R-PE (Cat. #FAB14001P, R&D Systems, Minneapolis, MN, USA), mouse anti human IL-12Rβ1-BB515 (CD212, BD Hori-zon, Cat. #565043, BD Biosciences, San Jose, CA, USA, and mouse anti human IL-12Rβ2-PerCP (Cat. #FAB1959C, R&D Systems). Data were acquired on a BD LSR Fortessa flow cytometer using the HTS plate reader and analyzed using FlowJo v10.6.2.

### 4.5. Flow Cytometry: Identification of RF/PF/IF and Receptor Detection in Intraclonal Fractions

PBMCs were thawed and stained for mouse anti human CD184 APC (CXCR4, Cat. #306510, BioLegend, San Diego, CA, USA), mouse anti human CD5 PE-Cy7 (Cat. #300622, BioLegend), and mouse anti human CD19 eFluor™ 450 (Cat. #48-0199-42, eBioscience, San Diego, CA, USA). For staining, cells (2 × 10^5^) in FACS buffer (PBS + 10% bovine serum albumin + 1% sodium azide) were incubated for 20 min at 4 °C and fixed with 0.1% formaldehyde in PBS. This staining enabled identification of three fractions: PF (CXCR4^dim^/CD5^bright^), RF (CXCR4^bright^/CD5^dim^), and IF (CXCR4^int^/CD5^int^). Within each fraction, expression of IL-23R, IL-12Rβ1, and IL-12Rβ2 was assessed as described above. In some experiments, multiparametric flow cytometry was performed using anti-human, REAfinity™ CD19 PE-Vio 770 (Cat. #130-113-647, Miltenyi, Bergisch Gladbach, Germany), mouse anti-human CD184 BV711 (CXCR4, Cat. #740799, BD Biosciences), mouse anti human CD5 BV421 (Cat. #562646, BD Biosciences), mouse anti human IL-23R-PE (Cat. # FAB14001P, Bio-techne/R&D systems), mouse anti-human IL-12Rβ1-APC (CD212, Cat. # 558708, BD Biosciences) and mouse anti human IL-12Rβ2-Alexa Fluor 488 (Cat. FAB1959G, Biotech-ne/R&D systems). Data were acquired on a CytoFLEX flow cytometer (Beckman coulter, Brea, CA, USA) and analyzed using FlowJo v 10.10.0.

### 4.6. Cell Sorting and RT-qPCR for Detection of IL-12Rβ1 RNA

Cell sorting was performed by staining PBMC from CLL samples with the following antibodies: mouse anti human CD19-FITC, mouse anti human CD3-PE-CY7, mouse anti human CD184-PE (CXCR4), mouse anti human CD5-APC (all from BD Biosciences). Resting (RF) and proliferative (PF) fractions were isolated from CD19 +CD5 + CLL cells after the exclusion of CD3+ T cells by a FACS ARIA II cell sorter (Becton Dickinson).

Total RNA was extracted from sorted cell populations using QIAzol Lysis Reagent (QIAGEN, Germantown, MD, USA, cat. no. 79306) and purified with the RNeasy Mini Kit (QIAGEN, cat. no. 74104). RNA concentration was measured using a NanoDrop 2000 spectrophotometer (Thermo Fisher Scientific, Waltham, MA, USA). A total of 50 ng RNA was reverse-transcribed and amplified in a single reaction using the Reliance One-Step Multiplex RT-qPCR Supermix kit (Bio-Rad, Hercules, CA, USA, cat. no. 12010220). Gene expression was assessed using TaqMan assays specific for human IL-12RB1 (Bio-Rad, qHsaCEP0057499) and HPRT1 (Bio-Rad, qHsaCIP0030549), used as the reference (housekeeping) gene. Relative mRNA levels were calculated using the ΔCt method and normalized to HPRT1.

### 4.7. Statistical Analysis

Statistical analyses were performed using GraphPad Prism (version 10.3.1). Comparisons between resting and proliferative fractions within the same patient were performed using paired non-parametric tests (two-sided Wilcoxon signed-rank test). Values are given as means ± SEM. A two-sided *p*-value < 0.05 was considered statistically significant.

## 5. Conclusions

This study shows that IL-12 family receptor expression is not uniform within CLL clones but is biased toward the proliferative fraction (PF; CXCR4^dim^/CD5^bright^), which is enriched for recently divided, tissue-emigrated cells. At baseline, PF displays higher IL-12Rβ1 at both surface and mRNA levels, and consequently a higher proportion of cells assembling IL-23R and (to a lesser extent) IL-12R receptor complexes.

Upon antigen-independent TLR9 activation, particularly with CpG + IL-15, CLL cells markedly upregulate IL-23R and IL-12Rβ1, while IL-12Rβ2 remains poorly inducible; this results in a preferential increase in the IL-23R complex over the IL-12R complex, with the strongest IL-23R complex induction occurring in PF relative to RF.

Overall, the data support an intraclonal functional skewing toward IL-23 responsiveness, highlighting the IL-23/IL-23R axis as a rational therapeutic vulnerability, while the limited inducibility of IL-12Rβ2 emerges as a key constraint potentially limiting IL-12–mediated tumor-suppressive signaling.

## Data Availability

The original contributions presented in this study are included in the article/Supplementary Materials. Further inquiries can be directed to the corresponding authors.

## Supplementary Materials

The following supporting information can be downloaded at https://www.mdpi.com/article/10.3390/ijms27031202/s1.

## Abbreviations

The following abbreviations are used in this manuscript:

BCR, B cell receptor; CLL, chronic lymphocytic leukemia; CpG, CpG oligodeoxynucleotides; FBS, fetal bovine serum; IF, intermediate fraction; IL, interleukin; mAb, monoclonal antibody; PB, peripheral blood; PBMCs, peripheral blood mononuclear cells; PBS, phosphate-buffered saline; PF, proliferative fraction; RF, resting fraction; RT-qPCR, reverse transcription quantitative polymerase chain reaction; TLR9, Toll-like receptor 9; 2-ME, 2-mercaptoethanol, SEM, standard error of the mean; CXCR4, C-X-C chemokine receptor type 4; FACS, fluorescence-activated cell sorting; APC, allophycocyanin; PE-Cy7, phycoerythrin-cyanine 7; OD, oligodeoxynucleotide; HTS, high-throughput sampler.
