# Supplementary material for: Intraclonal Enrichment of IL-23 Receptor Complex Expression in the Proliferative Fraction of Chronic Lymphocytic Leukemia
Source: Int J Mol Sci. 2026 Jan 25;27(3):1202. doi: 10.3390/ijms27031202 (PMC12898115; doi:10.3390/ijms27031202)
Supplement: Supplementary file 1 [file ijms-27-01202-s001.zip › ijms-4101003-supplementary.pdf]

Table S1. Features of CLL samples used in the study

ID	*IGHV*	FISH	Notch1	SF3B1	TP53
CA0202CLL1	M	Del(13q14) (100%)	WT	WT	WT
CD0310CLL1	UM	Del(13q14) (23.5% monoallelic; 67.5% biallelic)	WT	WT	WT
AC0120CLL1	M	+12 (96%); del(13q14) (22%)	WT	WT	WT
LG0038CLL1	UM	Del(11q22.3) (100%)	WT	WT	WT
LP0076CLL1	UM	Del(11q22.3)(28%); del(13q14) (80%)	WT	WT	WT
AA0396CLL1	UM	Del (13q14)(88%)	WT	WT	M (p.V157D)
BV0229CLL1	M	neg	WT	WT	WT
DG0186CLL1	M	neg	WT	WT	WT
DF0319CLL1	UM	+12 (93%)	WT	WT	WT
FS0257CLL1	UM	Del(11q22.3) (68%)	WT	WT	WT
PV0322CLL1	UM	Del(11q22.3)(100%); del(13q14) (100%)	WT	WT	WT
ET0128CLL1	UM	Del(13q14) (100%)	WT	WT	WT
BF0316CLL1	M	Del(13q14) (61% monoallelic; 31% biallelic)	M (delCT454)	WT	WT
DN0063CLL1	UM	Del(13q14) (100%)	M (delCT454)	WT	WT
CLL0239	M	Del(13q14) (82.5%)	NA	NA	NA
CLL0614	UM	NA	WT	WT	WT
CLL0850	M	NA	NA	NA	NA
CLL0957	UM	12 (84.5%)	WT	WT	WT
CLL1024	M	NA	WT	WT	WT
CLL1099	M	Del(13q14) (82.4%)	WT	WT	WT
CLL1127	M	Del(13q14) (86.%); del(14q32) (13.5%)	NA	NA	NA
CLL1413	M	12 (22.5%)	WT	MUT	WT
CLL1636	M	Del(13q14) (82%)	NA	NA	NA
CLL1808	UM	Del(13q14) (93%)	WT	WT	WT
CLL1867	UM	12 (35%)	NA	NA	NA
CLL1971	UM	12 (81%)	WT	WT	WT
CLL2107	UM	Del(13q14) (25.5%)	WT	WT	WT
CLL2147	M	NA	NA	NA	NA
